# Iatrogenic Non-Reconstructable Duodenal Injury Presenting without Peritonitis: An Indication of Pancreatoduodenectomy in the Presence of Safe Hands. A Case Report

**DOI:** 10.7759/cureus.1138

**Published:** 2017-04-06

**Authors:** Humaid Ahmad, Jahanzaib Haider, Sheeraz S Siddiqui, Sumbul Naz, Shams Nadeem Alam

**Affiliations:** 1 Department of Surgery / Hepatopancreatobiliary and Liver Transplant Unit, Dow University of Health Sciences (DUHS), Karachi, Pakistan; 2 Department of Surgery, Dow University of Health Sciences (DUHS), Karachi, Pakistan; 3 Hepatopancreatobiliary and Liver Transplant Unit, Dow University of Health Sciences (DUHS), Karachi, Pakistan

**Keywords:** pancreatoduodenectomy, duodenal injury, iatrogenic duodenal injuries, iatrogenic injury, post-nephrectomy complications

## Abstract

Iatrogenic duodenal injuries are rare complications of upper gastrointestinal endoscopic procedures, gallbladder, and right kidney operations. Management includes diverse options depending on a number of factors that include the size of the injury, timing of presentation, degree of peritoneal contamination, and presence of peritonitis and/or sepsis, etc. While most duodenal injuries are small, large complex non-reconstructable injuries may occasionally occur, requiring complex surgical procedures rather than primary repair which if done in the latter cases, may lead to further morbidity and mortality. Whipple’s pancreatoduodenectomy has rarely been performed for complex duodenal injuries especially in the iatrogenic setting. Here a case is reported of an iatrogenic duodenal injury that presented 12 days after a right open nephrectomy, with a dehisced right lumber incision having greater than one liter per day bilious discharge, for which Whipple’s pancreatoduodenectomy was performed. A discussion regarding factors which influenced per-operative decision making and probably led to a successful patient outcome is presented.

## Introduction

Duodenal injuries are uncommon in surgical practice [1]. Within this group, iatrogenic duodenal injury represents a rare but rising cause [2], which infrequently occurs as a complication of upper gastrointestinal endoscopic [3] and gallbladder procedures [4]. However, operations on the right kidney can also place the duodenum at risk [5]. During right kidney exposure, aggressive retraction or inadequate padding of retractors usually causes damage to the second part of duodenum [5]. While iatrogenic injuries may be small and amenable to primary repair [1, 5] or, more recently, to conservative/endoscopic management [6], large non-reconstructable duodenal defects (duodenal injuries of grade III and above according to The American Association for the Surgery of Trauma (AAST) Organ Injury Scale) can occur requiring more complex procedures [7-8].

Many procedures have been described for dealing with complex duodenal injuries [7]; however, there is no consensus on the ideal surgical option given any particular situation [2] with different schools of thought propagating the most effective surgical solution [7]. While different authors who reported iatrogenic duodenal injuries managed them differently with varying success [2-4], few authors have reported the results of pancreatoduodenectomy in this setting [7, 9-10], and to the authors’ knowledge, none such cases have been reported from their country of origin. In a recent study by Lissidini, et al. [7], out of 169 performed pancreatoduodenectomies, only two were for iatrogenic duodenal perforations. Although the indications of this procedure are not clearly defined [1], it seems only logical, if not imperative, to consider it as a last resort [1, 7], since it demands a longer operative time where the presence of greater surgical expertise can improve outcome [7].

The authors report a case of iatrogenic non-reconstructable duodenal injury presenting 12 days after right open nephrectomy for which pancreatoduodenectomy was performed with successful patient outcome. The per-operative rationale for resorting to such a radical option, and thus, the factors which probably contributed to this fruitful outcome are also discussed.

## Case presentation

A 25-year-old otherwise healthy female presented to emergency 12 days after a right open nephrectomy for a non-functioning kidney at another hospital. On the fourth postoperative day, she developed greenish discharge from her right flank incision. The patient later developed wound dehiscence with the discharge increasing to over one liter per day. She also became lethargic and developed spiking fevers. The primary surgeon’s referral notes revealed that duodenal injury had occurred during nephrectomy, for which primary repair was performed.

At presentation, the patient was in mild distress. She was anemic, dehydrated, and mildly jaundiced. On abdominal examination, there was copious amount of greenish discharge from a dehisced right-sided lumbar incision. The surrounding skin was excoriated. Tenderness was limited to the wound site with the abdomen being soft and non-tender indicating absence of peritonitis. Gut sounds and digital rectal examination were normal. Other systemic examinations were unremarkable, and save for the impending sepsis, the patient was relatively well-preserved. With the initial working diagnosis of suture-line leakage following an iatrogenic duodenal perforation repair, the patient was admitted for further diagnostic workup and supportive therapy.

The initial workup showed: Hemoglobin 8.1, potassium 3.4, total bilirubin 3.4, direct bilirubin 1.5, and alkaline phosphatase 190. The initial leucocyte count was 4200. Her platelet count, renal functions, coagulation profile, and other electrolytes were normal. Ultrasound showed bilateral mild pleural effusion with no free fluid in the abdomen.

Since the patient was neither in severe sepsis nor having generalized peritonitis, a decision was made to optimize the patient over a period of 48 hours, followed by exploration on the elective list. The pre-operative optimization plan included: complete bowel rest, central venous catheterization with total parenteral nutrition, and intravenous antibiotics. She was transfused two units of whole blood to correct her anemia.

**Figure 1:**
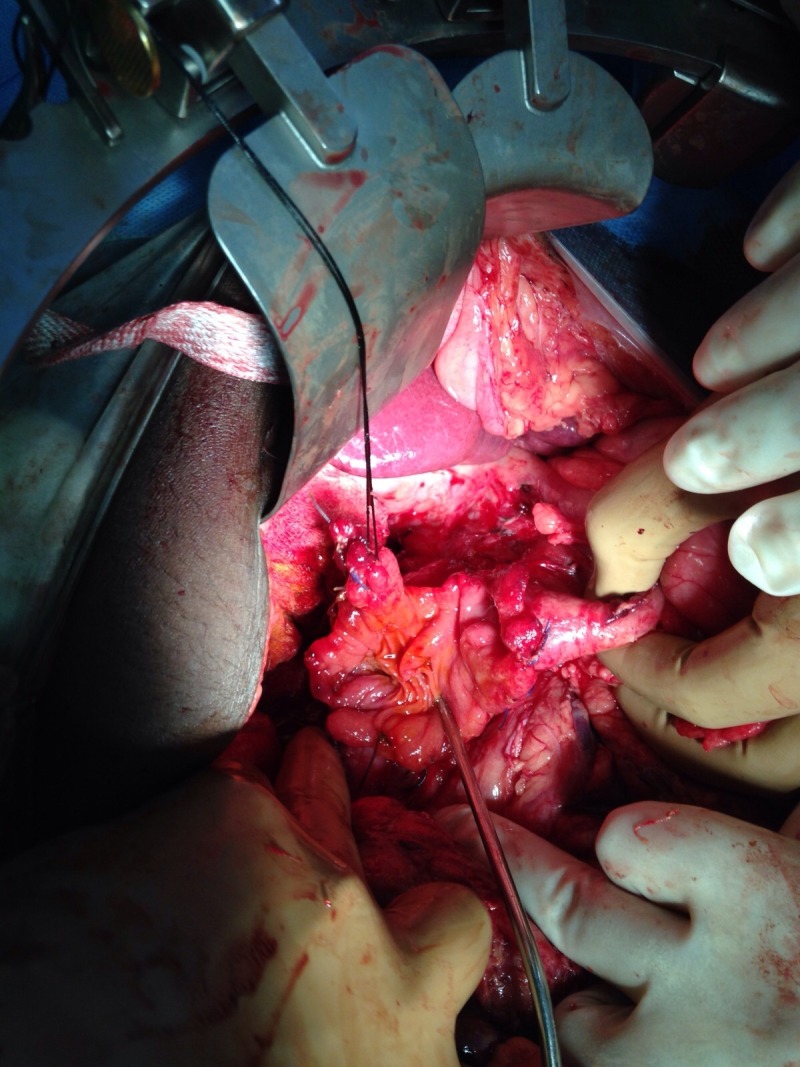
Peroperative picture showing large non-reconstructable duodenal injury

**Figure 2:**
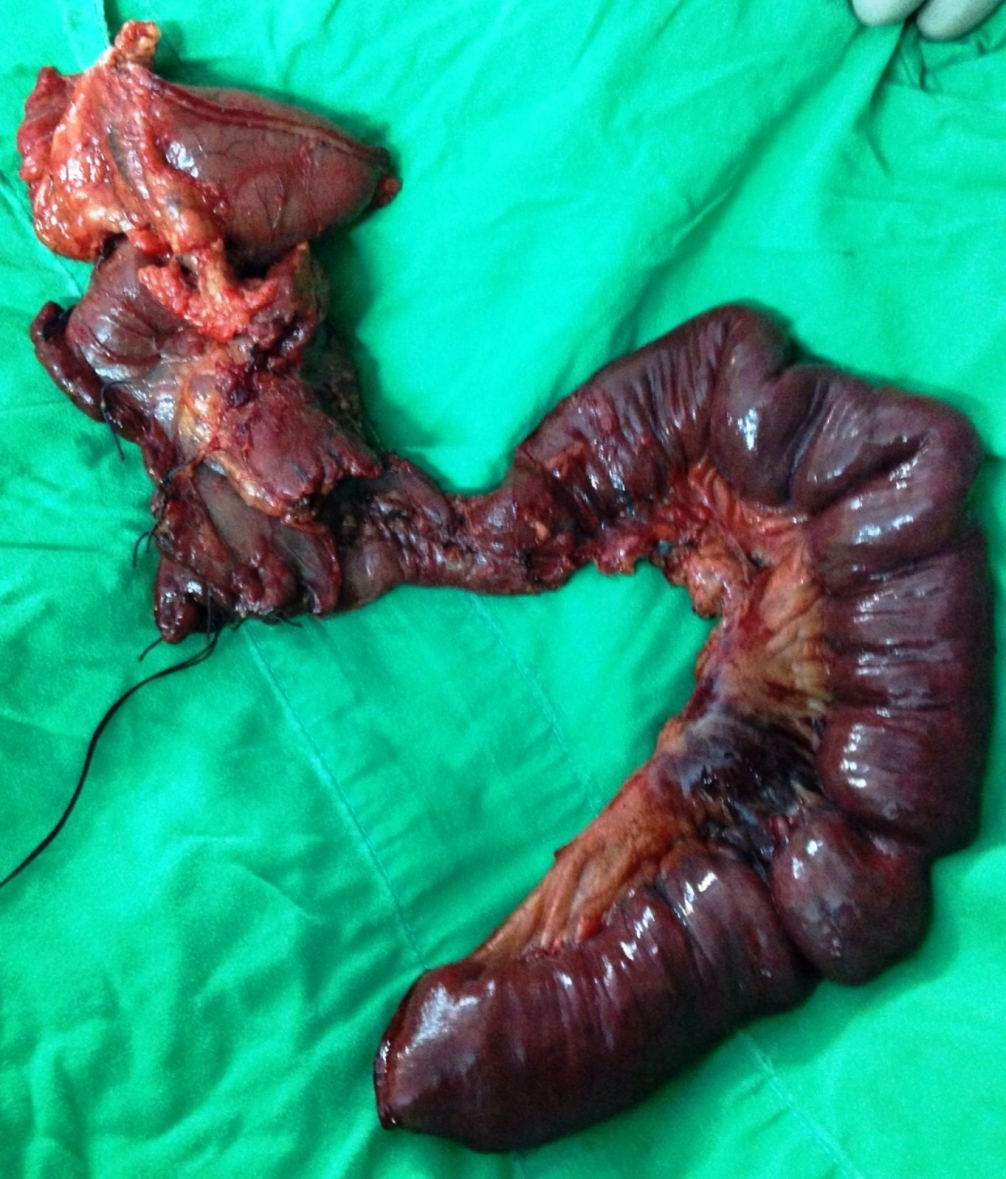
Whipple's pancreatoduodenectomy specimen showing large non-reconstructable duodenal injury

Exploratory laparotomy was performed as planned via midline incision and revealed an uncontaminated peritoneal cavity. The peritoneum covering the second part of the duodenum was thickened. Upon kocherization, the second part of duodenum was found to be edematous and inflamed with a large perforation where complete dehiscence of the previously repaired suture line had occurred. The thickened peritoneum and hepatic flexure of colon had walled off this perforation from the rest of the peritoneal cavity. The retroperitoneum in this area was found to be severely contaminated. Copious lavage was performed to clear the microbial load and protect the peritoneal space from cross-contamination. Dense adhesions between inferior vena cava and the duodenum were taken down, leading to a small iatrogenic tear of this vessel which necessitated its repair. Further duodenal dissection entailed a Cattell-Braasch maneuver. Once the whole duodenum had been mobilized and exposed, the second part was found to have lost more than 75% of the circumference wall (Grade IV according to AAST Organ Injury Scale), with the ampullary region being the only discernible part (Figures 1-2). Because the duodenum was deemed non-reconstructable by primary repair, different surgical options were considered on the table.

As the duodenum was not healthy enough to take suture for anastomosis even after freshening of the perforation edges and the small bowel was completely healthy consequent to contamination being limited to the retroperitoneum, Whipple’s pancreatoduodenectomy was considered the most feasible option. The involvement of a trained hepatopancreatobiliary surgeon in the case made this decision easier. The procedure was performed in standard manner with pancreatojejunostomy being constructed in an end-to-end invaginated fashion.

The patient tolerated the prolonged surgery well and was shifted to intensive care unit for one day. Postoperatively, she remained stable and was orally allowed on her fifth postoperative day. She developed a small amount of seropurulent discharge from the upper part of her midline wound on her 21st postoperative day. Culture and sensitivity of the fluid revealed no growth and amylase content was normal. A fistulogram and computed tomography (CT) scan were performed, which detected a small intraperitoneal collection but no pancreatic and/or biliary fistula. The discharge gradually decreased with the institution of broad-spectrum antibiotic therapy and she was discharged on her 33rd postoperative day at which time she was completely tolerating a full diet and her seropurulent discharge had completely resolved.

## Discussion

Iatrogenic duodenal injuries due to right kidney procedures are established complications [5]. Since there is a relative paucity of references specifically describing surgical interventions for iatrogenic duodenal injuries [3] and standard procedures have not yet been established [2], current literature search reveals that where surgery is necessary, surgeons tend to use guidelines described for traumatic duodenal injuries to deal with these rare situations. Regardless of the management plan being employed, the outcome depends mainly on the size of duodenal defect, timing of presentation (duration of injury), degree of peritoneal contamination, presence of generalized peritonitis, and/or sepsis [1, 7].

With injuries having large duodenal defects, attempting primary repairs can clearly predispose to anastomotic dehiscence and prolonged morbidity later on [2]. In order to forestall such consequences, a variety of more complex surgical techniques has been evolved such as duodenojejunostomy, serosal patch repairs, primary repair with pyloric exclusion, etc. [1]. However even for these procedures, results are controversial [1] and integrity of repair, among other factors, depends on the presence of healthy tissues able to take and hold the suture. Such a situation seldom exists with delayed presentation, which is the usual situation in cases of iatrogenic injuries [6]; the reason being that delay allows extensive edema and inflammation of the duodenal wall to set in [2, 6]. This phenomenon partly occurs due to the enzyme-rich duodenal fluid leaking over the edges of the duodenal perforation [2]. The resultant autodigestive process also damages surrounding organs [2] with all these factors increasing the possibility of post-procedure anastomotic dehiscence [2].

In this case, the duodenal perforation was not only very large but the remaining duodenal wall was also very edematous, friable, and inflamed. In comparison, the small bowel was healthy as a consequence of the peritoneal cavity being spared from contamination. While duodenojejunostomy or serosal patch repairs were considered, it was decided that such unhealthy duodenal wall would not take up suture despite freshening of perforation edges and the assured anastomotic dehiscence post-procedure would then cause contamination of the peritoneal cavity, hitherto clean and sterile; thus resulting in a worsened outcome. This deliberation along with the presence of healthy non-edematous jejunum facilitated the decision of pancreatoduodenectomy as the procedure of choice for this patient; a procedure where healthy non-edematous jejunum would be desirable to perform the highly technical anastomoses associated with the procedure [8]. While spillage of duodenal contents out of the dehisced lumbar incision with resultant limited severity of sepsis was another favorable factor considered, the most important reason probably influencing decision making was the involvement of a trained hepatopancreatobiliary surgeon in the case, the presence of whom during the decision making process and surgery has been related to better outcomes of pancreatoduodenectomy [7-8]. It has even been advocated that general surgeons dealing with such milieu of injuries gain experience in hepatopancreatobiliary surgery as this highly complex procedure requires well-developed skills to perform [7].

As is evidenced from this case, the delayed presentation may not be as important a factor in determining the outcome if other factors are controlled. Lastly, another factor that we feel definitively led to patient survival was the decision to not rush this patient to surgery in emergency, but to choose a plan of optimization and surgery in a more favorable setting, a decision guided by a thorough clinical evaluation of the patient at the time of presentation.

This case represents one of the early cases in our experience of hepatopancreatobiliary surgery and Whipple’s pancreatoduodenectomy.

## Conclusions

We propose that pancreatoduodenectomy, usually considered a last surgical option, may have more place in cases of iatrogenic non-reconstructable duodenal injuries if the peritoneal cavity has been spared from contamination, the small bowel is healthy, and the patient is not in severe sepsis, especially if safe hands are available; the main reason being that these injuries will usually not have a duodenal wall healthy enough to bear repairs of other proposed procedures with increased chances of post-procedure leakage and further morbidity/mortality. As such, general surgeons working in tertiary care hospitals dealing with such situations, in addition to other techniques, should also have this procedure in their armamentarium in order to adapt to any given situation. A careful literature search reveals that authors who reported similar situations in the past have drawn, more or less, similar conclusions.
